# Limosilactobacillus fermentum Limits Candida glabrata Growth by Ergosterol Depletion

**DOI:** 10.1128/spectrum.03326-22

**Published:** 2023-02-21

**Authors:** Isabella Zangl, Reinhard Beyer, Arianna Gattesco, Roman Labuda, Ildiko-Julia Pap, Joseph Strauss, Christoph Schüller

**Affiliations:** a University of Natural Resources and Life Sciences, Vienna, Institute of Microbial Genetics, Tulln, Austria; b Institute of Food Safety, Food Technology and Veterinary Public Health, University of Veterinary Medicine Vienna, Vienna, Austria; c Bioactive Microbial Metabolites, University of Natural Resources and Life Sciences, Vienna (BOKU), Institute of Microbial Genetics, Tulln, Austria; d University Hospital of St. Pölten, Institute for Hygiene and Microbiology, St. Pölten, Austria; e Core Facility Bioactive Molecules: Screening and Analysis, University of Natural Resources and Life Sciences, Vienna, Austria; Septomics Research Center, Friedrich Schiller University and Leibniz Institute for Natural Product Research and Infection Biology—Hans Knöll Institute

**Keywords:** *Candida glabrata*, *Lactobacillus*, candidiasis, ergosterol, microbial communities, probiotics, stress response, transcriptional regulation

## Abstract

Candida glabrata is a human-associated opportunistic fungal pathogen. It shares its niche with *Lactobacillus* spp. in the gastrointestinal and vaginal tract. In fact, *Lactobacillus* species are thought to competitively prevent *Candida* overgrowth. We investigated the molecular aspects of this antifungal effect by analyzing the interaction of C. glabrata strains with Limosilactobacillus fermentum. From a collection of clinical C. glabrata isolates, we identified strains with different sensitivities to *L. fermentum* in coculture. We analyzed the variation of their expression pattern to isolate the specific response to *L. fermentum*. C. glabrata-*L. fermentum* coculture induced genes associated with ergosterol biosynthesis, weak acid stress, and drug/chemical stress. *L. fermentum* coculture depleted C. glabrata ergosterol. The reduction of ergosterol was dependent on the *Lactobacillus* species, even in coculture with different *Candida* species. We found a similar ergosterol-depleting effect with other lactobacillus strains (Lactobacillus crispatus and Lactobacillus rhamosus) on Candida albicans, Candida tropicalis, and Candida krusei. The addition of ergosterol improved C. glabrata growth in the coculture. Blocking ergosterol synthesis with fluconazole increased the susceptibility against *L. fermentum*, which was again mitigated by the addition of ergosterol. In accordance, a C. glabrata *Δerg11* mutant, defective in ergosterol biosynthesis, was highly sensitive to *L. fermentum*. In conclusion, our analysis indicates an unexpected direct function of ergosterol for C. glabrata proliferation in coculture with *L. fermentum*.

**IMPORTANCE** The yeast Candida glabrata, an opportunistic fungal pathogen, and the bacterium Limosilactobacillus fermentum both inhabit the human gastrointestinal and vaginal tract. *Lactobacillus* species, belonging to the healthy human microbiome, are thought to prevent C. glabrata infections. We investigated the antifungal effect of Limosilactobacillus fermentum on C. glabrata strains quantitively *in vitro*. The interaction between C. glabrata and *L. fermentum* evokes an upregulation of genes required for the synthesis of ergosterol, a sterol constituent of the fungal plasma membrane. We found a dramatic reduction of ergosterol in C. glabrata when it was exposed to *L. fermentum*. This effect extended to other *Candida* species and other *Lactobacillus* species. Furthermore, fungal growth was efficiently suppressed by a combination of *L. fermentum* and fluconazole, an antifungal drug which inhibits ergosterol synthesis. Thus, fungal ergosterol is a key metabolite for the suppression of C. glabrata by *L. fermentum*.

## INTRODUCTION

Candida glabrata (also Nakaseomyces glabrata) and *Lactobacillus* spp. both belong to the human microbiome (1, 2). C. glabrata is part of the *Nakaseomyces* clade and is an opportunistic pathogen, which habitats the human oral, gastrointestinal, and vaginal tract (3, 4). It is the second most prevalent cause, after Candida albicans, of vulvovaginal candidiasis (VVC), an acute inflammatory disease of the vaginal tract (5). Despite both *Candida* spp. causing infections, C. glabrata is more closely related to Saccharomyces cerevisiae than to C. albicans (6).

Vulvovaginal candidiasis is traditionally treated with azoles, inhibiting the cytochrome P450 enzyme lanosterol demethylase (14α-demethylase), encoded by *ERG11*, in the ergosterol biosynthesis pathway (7). Ergosterol is an essential part of the fungal cell membrane, and inhibition of the ergosterol biosynthesis pathway by azoles arrests growth (8). However, in contrast to C. albicans, C. glabrata isolates often possess intrinsic resistance against azoles (9). Resistance against azoles in C. glabrata is dependent partly on the overexpression of drug-exporting membrane pumps of the ABC transporter class (8).

*Lactobacillus* spp. are part of the healthy human microbiome and inhabit mainly the gut and vaginal tract (10). Whereas the gut microbiome is rather diverse, in the vaginal tract, lactobacilli are the predominant species (11). The most dominant *Lactobacillus* spp. in the vaginal tract are Lactobacillus crispatus, Lactobacillus gasseri, Lactobacillus jensenii, and Lactobacillus iners (11). Interestingly, a decrease of L. crispatus in combination with an increase of *L. iners* is associated with VVC (12). *Lactobacillus* spp. in general are thought to prevent *Candida* infections by reducing the adhesion of the fungus to the mucosa and producing favorable metabolites, for example, lactic acid and acetic acid, as well as enhancing the epithelial cell immune defense mechanisms (13–15). Limosilactobacillus fermentum (formerly Lactobacillus fermentum [16]) can be isolated from plants and spontaneously fermented cereals and is taken up by consumption on a regular basis. However, it is not adapted to the human gastrointestinal tract (16). Since *L. fermentum* is isolated from human feces on a regular basis, it is regarded as part of a healthy human gut microbiome (1, 16, 17). *L. fermentum* is considered a generally recognized as safe (GRAS) species and is often utilized as a probiotic. Furthermore, *L. fermentum* was shown to have an antifungal effect against Candida albicans and Candida glabrata (18, 19). *L. fermentum* is able to metabolize cholesterol (20, 21), the ergosterol equivalent of the human cell. In the human body, lactobacilli were able to regulate cholesterol content by regulating the expression of genes involved in cholesterol synthesis, metabolism, transport, and absorption (22).

The molecular mechanisms behind the *Lactobacillus*-induced antifungal effects are still unclear. The interaction of C. albicans with Lactobacillus rhamnosus (strain GG) had a global effect on virulence mechanisms, such as adhesion and change of morphology, and notably leads to downregulation of genes for ergosterol biosynthesis (23, 24). The interaction between C. albicans and Lactobacillus rhamnosus leads to decreased pathogenicity of the fungus. Pretreatment of intestinal epithelial cells with L. rhamnosus leads to a more hostile environment for C. albicans, forcing a metabolic shift, which is accompanied by a decreased virulence (25). The C. glabrata-*Lactobacillus* interaction has so far been linked to the mitogen-activated protein (MAP) kinase Hog1, which has a conserved role in osmotic stress response. C. glabrata Hog1 is involved in response to short-chain weak organic acids, such as hexadienoic acid, which is also known as sorbic acid. The pathway was found to be crucial for lactic acid response at a physiological level (110 mM) (26). C. glabrata Hog1 regulated the osmotic stress response pathway that is also involved in membrane homeostasis. Furthermore, the presence of *Lactobacillus* lead to a decrease of the C. glabrata adhesion regulating gene *YAK1* and the Yak1-dependent adhesin gene *EPA6* (27).

In this study, we investigated the interaction between *L. fermentum* and clinical C. glabrata isolates in coculture. We used a quantitative screening method to quantify the growth-inhibiting effect of *L. fermentum* and explored the differences between individual C. glabrata isolates. We analyzed the gene expression differences between clinical isolates during coculture and show that C. glabrata requires ergosterol biosynthesis for survival during coculture with *L. fermentu*m.

## RESULTS

### Candida glabrata isolates show phenotypic variability during coculture conditions.

**FIG 1:**
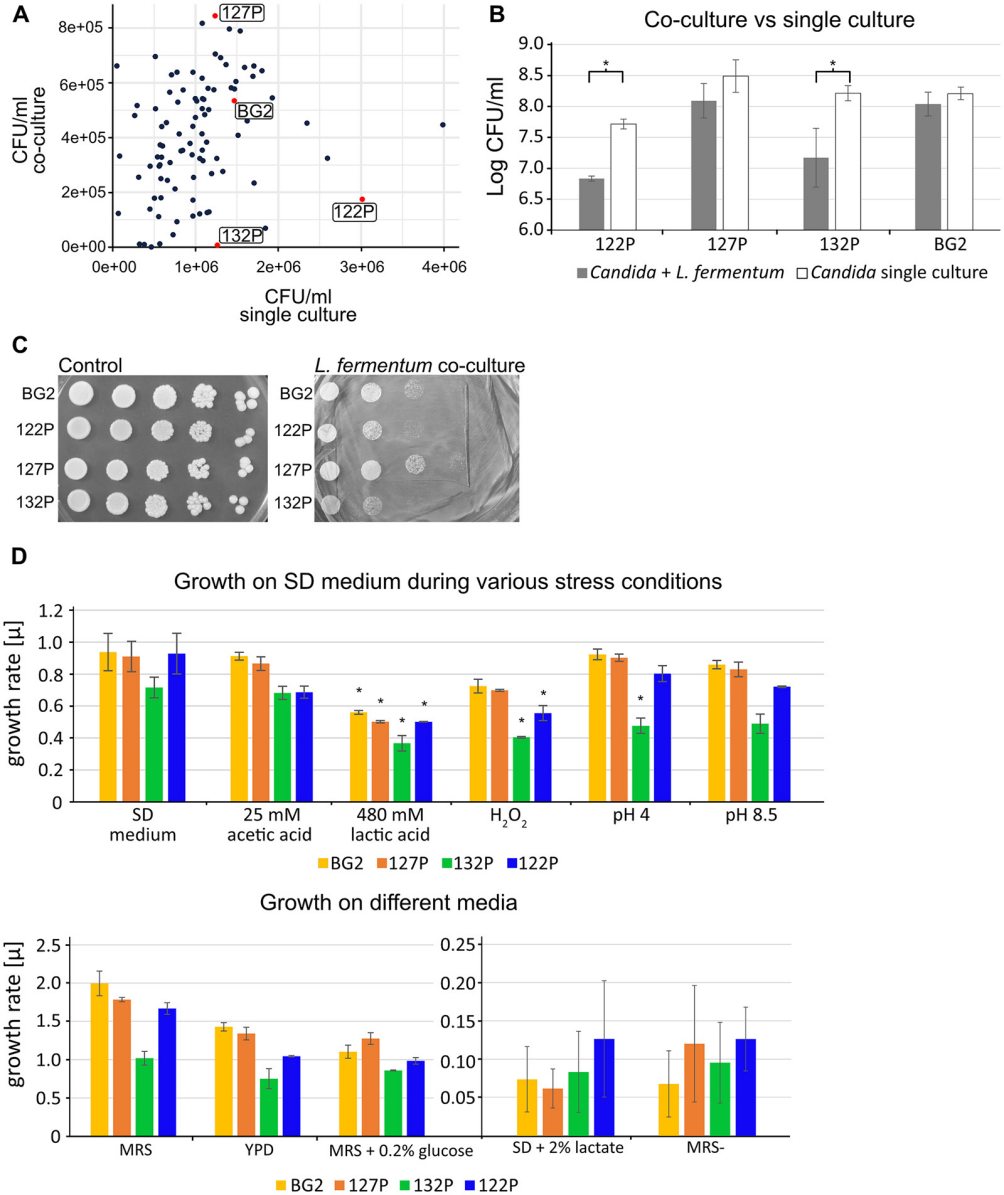
*L. fermentum* exerts an antifungal effect against Candida glabrata. (A) Determined CFU/mL of Candida glabrata and *L. fermentum* coculture versus C. glabrata single culture after 20 h; each data point represents at least two biological replicates (*n* = 2 to 3), and red datapoints highlight isolates which were picked for further analysis. (B) Counted log CFU/mL of Candida glabrata and *L. fermentum* coculture versus counted CFU/mL of single *Candida* culture after 24 h; data represent mean log CFU/mL of at least three biological replicates (*n* = 3 to 5). Asterisks represents a statistical difference between single and coculture conditions (*, *P* ≤ 0.05). (C) Candida glabrata susceptibility against *L. fermentum* on MRS medium. Isolates were spotted in serial dilution onto MRS or MRS with 50 μL of *L. fermentum* culture (OD of 1). (D) Quantitative analysis of growth performance during different conditions and stressors; data represent calculated growth rates after 24 h. Error bars represent the standard error of at least two biological replicates (*n* = 2 to 4). Asterisks represent a statistical difference between growth rate in SD medium (=control) and growth rate during stress (*, *P* ≤ 0.05).

*Lactobacillus* spp. exert an antifungal effect on C. glabrata *in vitro* (18, 28). To investigate this effect, we performed a quantitative analysis of the growth behavior of 94 C. glabrata isolates in coculture with *L. fermentum* (Fig. 1A). We assessed the growth performance of each strain by determining CFU/mL after 20 h of incubation in coculture and in a single-culture setting. We found that the presence of *L. fermentum* leads to a significant decrease of *Candida* growth. However, this effect is strain specific, as not all *Candida* strains were influenced to the same extent. For further analysis we chose the laboratory strain BG2 (29) and 3 clinical isolates (127P, 132P, and 122P) and counted CFU/mL after 24 h (Fig. 1B). The C. glabrata isolates can be grouped into resistant (BG2 and 127P) and sensitive (122P and 132P) to *L. fermentum*. The growth performance of isolates against *L. fermentum* on solid medium revealed the same clustering as in liquid, although some growth reduction could be observed for all isolates (Fig. 1C). Next, we investigated phenotypic traits, such as general stress susceptibility of the isolates, to assess a possible connection to *L. fermentum* susceptibility (Fig. 1D). We concentrated on conditions associated with *L. fermentum* stress, for example exposure to lactic and acetic acid or low glucose levels. We quantitatively determined the growth rates of the isolates under these conditions. Strain 132P had the lowest growth rate during various stress tests. The isolates clustered in the same groups as in the *L. fermentum* sensitivity test, namely, generally lower growth rates (122P and 132P) and generally higher growth rates (BG2 and 127P) (Fig. 1C), except for growth in yeast extract-peptone (YP) medium with 2% lactate or no added carbon source, where all isolates behaved similarly. The same pattern was observed for control conditions (YP-dextrose [YPD], Sabouraud dextrose [SD], or MRS medium), which indicates an overall slower growth of 122P and 132P than that of the other two strains. H_2_O_2_ stress was the only condition which led to significant growth reduction in the *L. fermentum* sensitive strains but not in the resistant C. glabrata isolates.

### Limosilactobacillus fermentum causes a shift in the Candida glabrata transcriptome.

**FIG 2:**
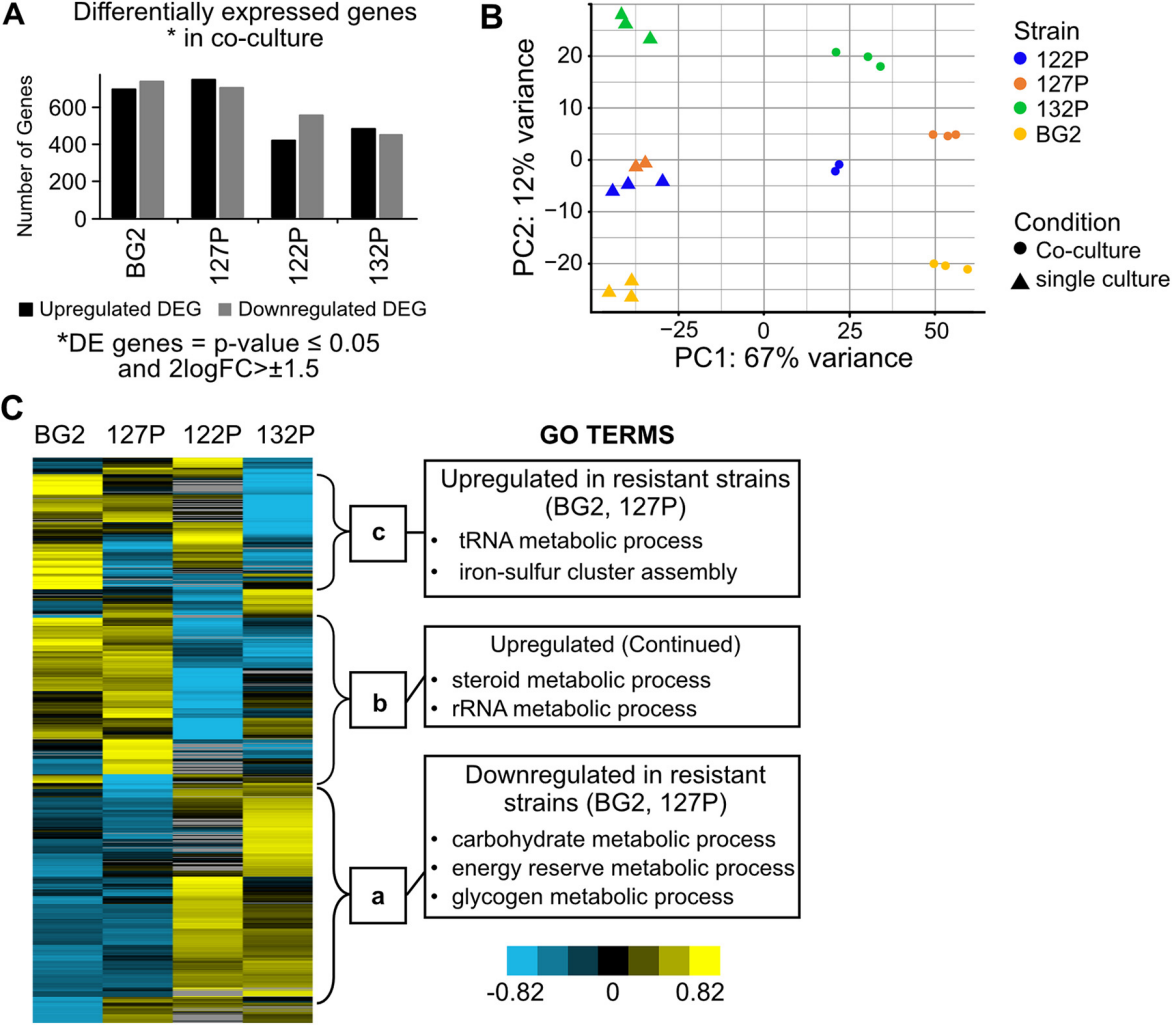
Transcriptional response of four *Candida* isolates in coculture with *L. fermentum*. (A) Number of significantly up- and downregulated differentially expressed genes (DEGs) (*P* ≤ 0.05; minimum log2 fold change [FC], ≥1.5) (*n* = 2 to 3). (B) Principal component analysis of the isolates and conditions; strains are displayed in different colors; conditions are displayed in different shapes. (C) Heatmap for a comparison of the gene expression of the *Lactobacillus*-resistant and -sensitive isolates during coculture; data represent log2FC after being filtered (cutoff log2FC, ≥2; *P* ≤ 0.05; and basecount, >20), normalized, and centered. Yellow shows upregulation, blue shows downregulation, and a to c represent clusters. The GO terms table shows the most important terms in cluster; Table S4 contains the full list of GO Terms.

We used transcriptome sequencing (RNA-seq) to investigate how the presence of *L. fermentum* changes the gene expression pattern of C. glabrata. We compared the expression profiles of a *Candida* culture with a *L. fermentum*-*Candida* coculture after 10 h of incubation. In total, more significantly downregulated differentially expressed genes than upregulated differentially expressed genes (Fig. 2A) were detected. More differentially expressed genes were found in the two *L. fermentum*-resistant isolates (BG2 and 127P) than those in the two sensitive isolates (122P and 132P). Principal component analysis visualized the differences between the isolates (Fig. 2B). We conclude that coculture leads to a transcription shift in all C. glabrata isolates and is the main reason for variance (67%) in the samples. Clustering of single-culture profiles points to a similar gene expression pattern under control conditions. PC2 shows the similarity between the different isolates. However, PC2 accounts for only 12% of the total variance. Therefore, the difference of transcriptome profiles between isolates is small compared with the effect caused by different culture conditions.

Looking closer at the differences between the resistant and sensitive isolates, we found a set of genes which are repressed in the *Lactobacillus*-resistant isolates BG2 and 127P and induced in the *Lactobacillus*-sensitive isolates (Fig. 2C). Gene ontology (GO) enrichment analysis of the expression data shows genes associated with carbohydrate metabolism and energy reserve (Fig. 2C; see Table S4 in the supplemental material). Repression of energy reserve metabolism hints that the resistant isolates are not restricting growth and are less bothered by the presence of *Lactobacillus* than sensitive ones. In the induced gene set, the separation between resistant and sensitive C. glabrata isolates was less clear. In general, we found genes related to steroid metabolism, rRNA metabolism, and iron-sulfur cluster assembly to be upregulated (Fig. 2C; Table S4).

### Interaction with *L. fermentum* leads to the upregulation of ergosterol synthesis.

**FIG 3:**
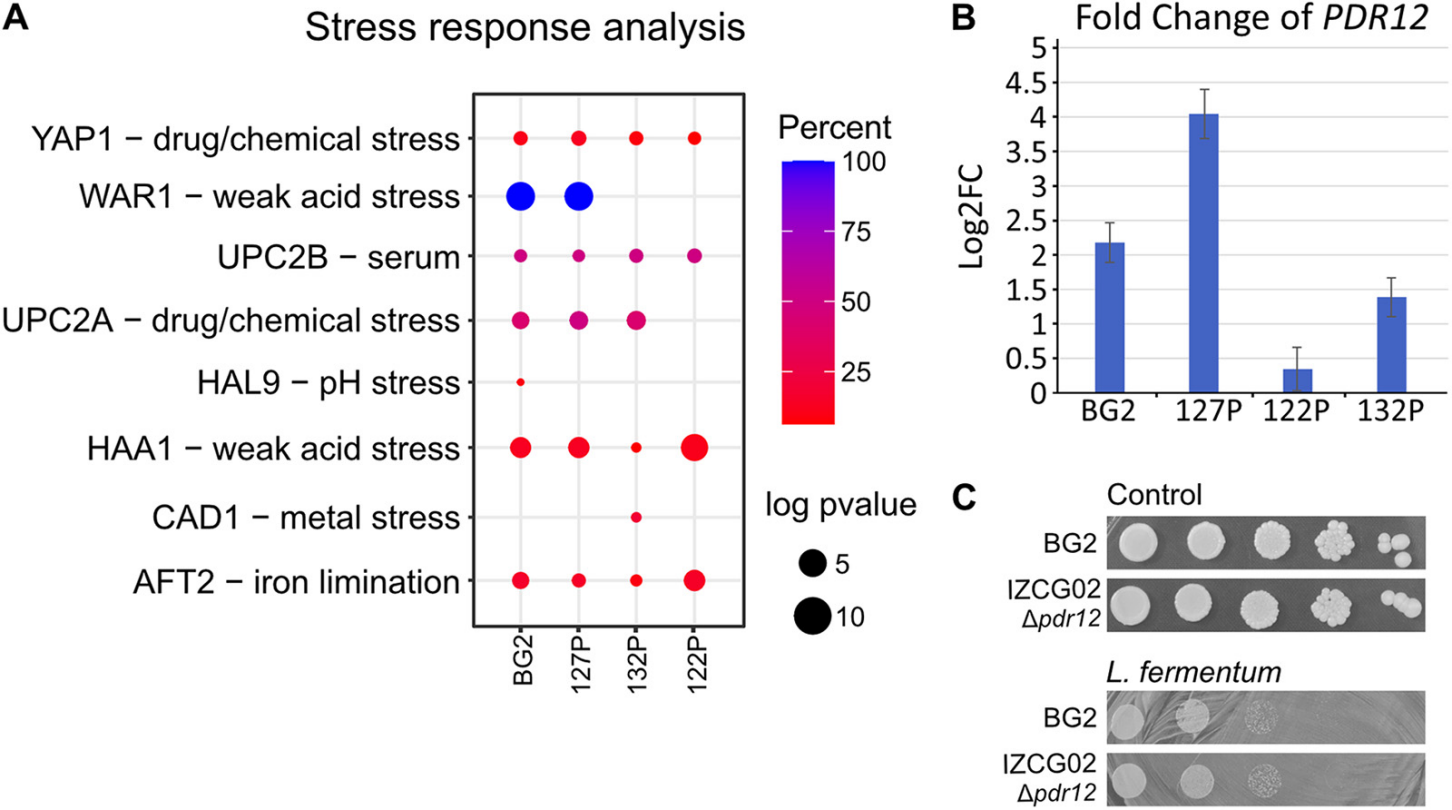
Enrichment analysis of expression data. (A) Clustering of upregulated DEG according to common transcription factor (TG) using the PathoYeastract page; percent represents the percentage of detected DEGs in the total amount of targets of the TF. (B) Log2FC of *PDR12* during RNA-seq. Error bars represent the standard error of the logfoldchange (lfcSE) of *PDR12* (*n* = 2 to 3). (C) Serial dilutions of BG2 and IZCG02 (Δ*pdr12*) on plates with *L. fermentum* (50 μL OD_600_ of 1 per plate).

Clustering using the PathoYeastract approach (30, 31) showed that genes regulated by transcription factors were associated with weak acid stress, drug/chemical stress, and iron limitation (Fig. 3A). Weak acid stress was to be expected since *L. fermentum* is producing acetic and lactic acid. The transcription factor *Haa1* is involved in acetic acid stress (32). The gene expression patterns of the *Haa1* regulon, as well as acetate-grown C. glabrata, did not reveal significant similarities to our data (see Fig. S1A and B in the supplemental material). Acetate stress may therefore be ruled out as a reason behind the transcriptome shift in coculture.

In yeast, *PDR12* is required for weak acid resistance (33–35) and is the single gene regulated by the weak acid response transcription factor War1. *CgPDR12* was upregulated in the *Lactobacillus*-resistant isolates (Fig. 3B). However, a Δ*pdr12* knockout mutant was not sensitive to *L. fermentum* (Fig. 3C). Therefore, a substantial role during *Lactobacillus* interaction is unlikely for *PDR12*.

Lactobacilli are able to produce H_2_O_2_ and thus may cause oxidative stress. Although the downregulated genes during oxidative stress overlapped with our data set, the upregulated genes were not similar (Fig. S1C). Therefore, the production of H_2_O_2_ is unlikely to lead to the observed transcriptomic shift in gene expression.

Response to antifungal drug stress, for example azoles, often involves ergosterol synthesis. Ergosterol is an essential part of fungal cell membrane and is used as a target for antifungals. Interestingly, almost all genes of the ergosterol biosynthesis pathway were found upregulated in all C. glabrata isolates (see Fig. S2 in the supplemental material). Upregulated genes were related to the import and regulation of ergosterol synthesis, such as *UPC2A* and *UPC2B*, which are transcription factors responsible for sterol biosynthesis (36), *AUS1* responsible for sterol import (37), and *HES1* involved in C. glabrata sterol biosynthesis during azole stress (38).

Ergosterol plays a role in response to hypoxia and azole antifungals (39). The coculture setting might cause limiting oxygen availability due to the presence of lactobacilli which leads to an increase of ergosterol biosynthesis. However, we think this process is unlikely because our data do not show an induction of gene sets of the general hypoxia pathways derived from S. cerevisiae (see Fig. S3B in the supplemental material). We further found no differences in the oxygen content of the medium after 10 h of incubation and between coculture conditions and single-culture conditions (Fig. S3A), thus rejecting a potential hypoxia response in our experiment.

**FIG 4:**
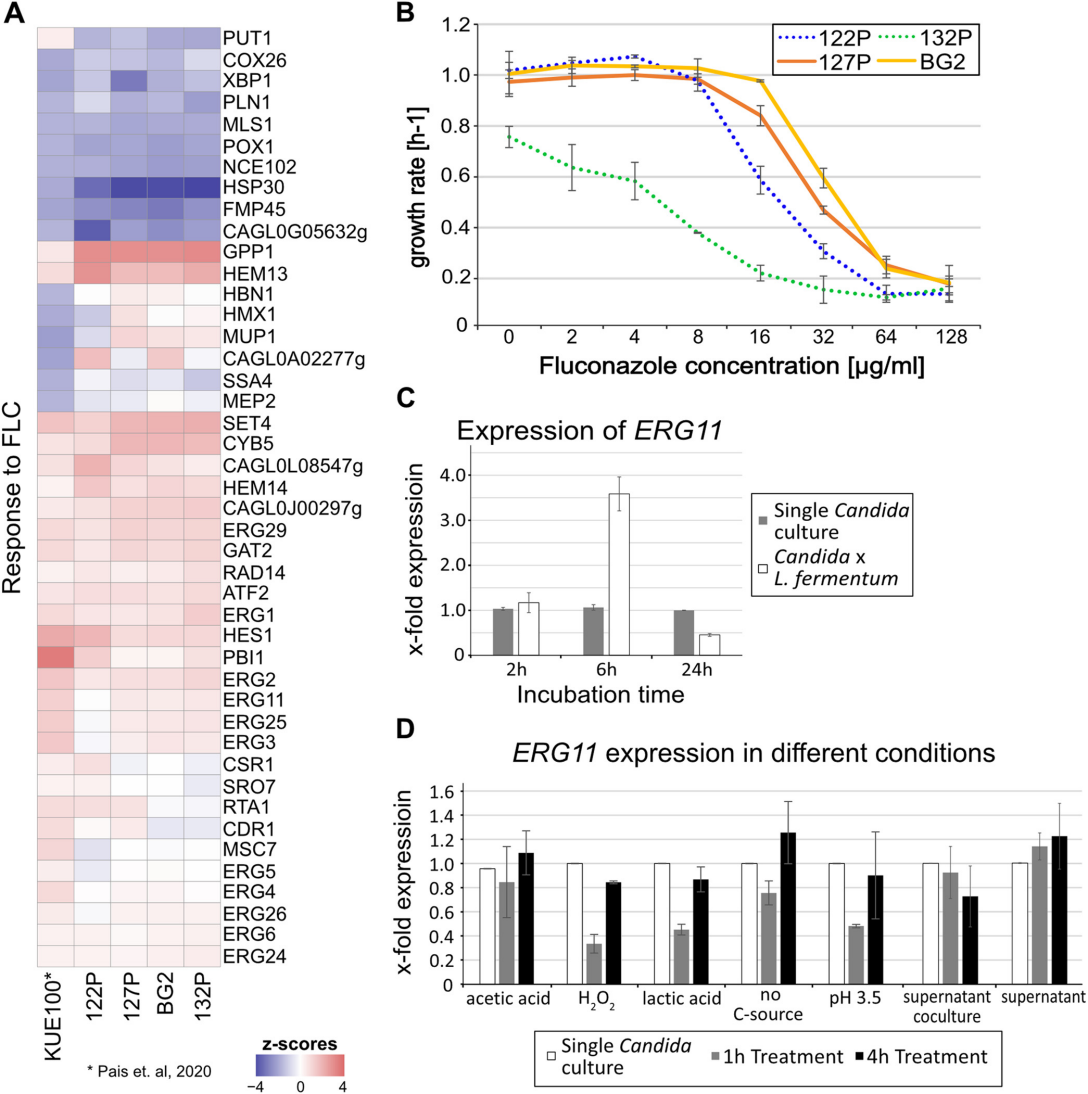
Fluconazole response shares similarities to *Lactobacillus* response. (A) Heatmap of expression data of mild FLC stress compared with the *Lactobacillus* response of the tested isolates; Z-scores represent log2FC scaled to the mean of the column (red, upregulation; blue, downregulation). (B) Growth rates of the C. glabrata isolates during different concentrations of FLC; each data point represents he mean growth rate of two biological replicates (*n* = 2). (C) Expression data of *ERG11* during coculture and single culture after 2 h, 6 h, and 24 h of cultivation; *n* = 2. (D) Expression data of *ERG11* of BG2 treated with different stressors after 1 h (immediate response) and 4 h of treatment; supernatants were taken after 24 h of incubation; expression data were normalized to *ACT1*; *n* = 2.

Azole antifungals inhibit cytochrome p450 lanosterol C14α demethylase encoded by *ERG11*. Deletion of *ERG11* prevents ergosterol biosynthesis, leading to an increase of toxic sterols which are fungistatic (8). Fluconazole (FLC) treatment leads to an increase of *ERG11* expression in C. glabrata (40). We therefore compared the coculture expression profile data to the gene expression pattern characteristic for fluconazole treatment. Indeed, we found that the expression pattern for fluconazole stress in the strain KUE100 is very similar to the coculture profile (Fig. 4A). C. glabrata isolates are more resistant to fluconazole than C. albicans (9, 41). We determined the MIC at 50% growth inhibition (MIC_50_) of the isolates by measuring the optical density over time and calculating the respective growth rates (Fig. 4B). Fluconazole up to 128 μg/mL reduced the growth rate of all isolates significantly, but it was not fungicidal. The *L. fermentum*-resistant strains BG2 and 127P had the highest resistance level with an MIC_50_ of 32 μg/mL fluconazole, followed by 122P (16 μg/mL) and 132P (8 μg/mL). Thus, the fluconazole resistance profile mirrors that seen with resistance to *L. fermentum*. Also considering the overlap in gene expression, the findings suggest effective similarities between both conditions for C. glabrata.

### C. glabrata ergosterol biosynthesis is not triggered by a simple metabolite.

To evaluate the time-dependent effect of the coculture condition, we measured *ERG11* expression via quantitative PCR (qPCR) in the BG2 strain (Fig. 4C). *ERG11* expression levels peaked after 6 h and was still increased after 10 h of coincubation. After a 24-h incubation, the *ERG11* expression level was similar to untreated cultures. This finding suggests an accumulative effect due to an increased biomass of *L. fermentum* rather than an immediate stress effect. We also explored the following conditions likely caused by *L. fermentum* exposure: acetic acid, lactic acid, H_2_O_2_, acidic pH (pH 3.5), and glucose depletion (Fig. 4D). We used a 1-h treatment for the immediate effect and a 4-h treatment for the delayed effect. As expected, we found a drop in *ERG11* expression level as an immediate effect possibly due to stress-induced transient growth inhibition. After the 4-h treatment, *ERG11* expression returned to unstressed levels, suggesting that the tested conditions might not play a decisive role in the upregulation of ergosterol biosynthesis. However, during coculture, a continuous mixture of stressors would be closer to the *in vivo* situation. To identify a compound or a compound mixture secreted by *L. fermentum*, we added the culture supernatant and coculture supernatant. A carbon source was added to prevent a starvation response. Both cell-free supernatants did not lead to a significant increase of *ERG11*. Taken together, we conclude from these experiments that the vicinity of living *L. fermentum* cells inhibits C. glabrata growth.

### Ergosterol availability is crucial for C. glabrata growth in coculture.

**FIG 5:**
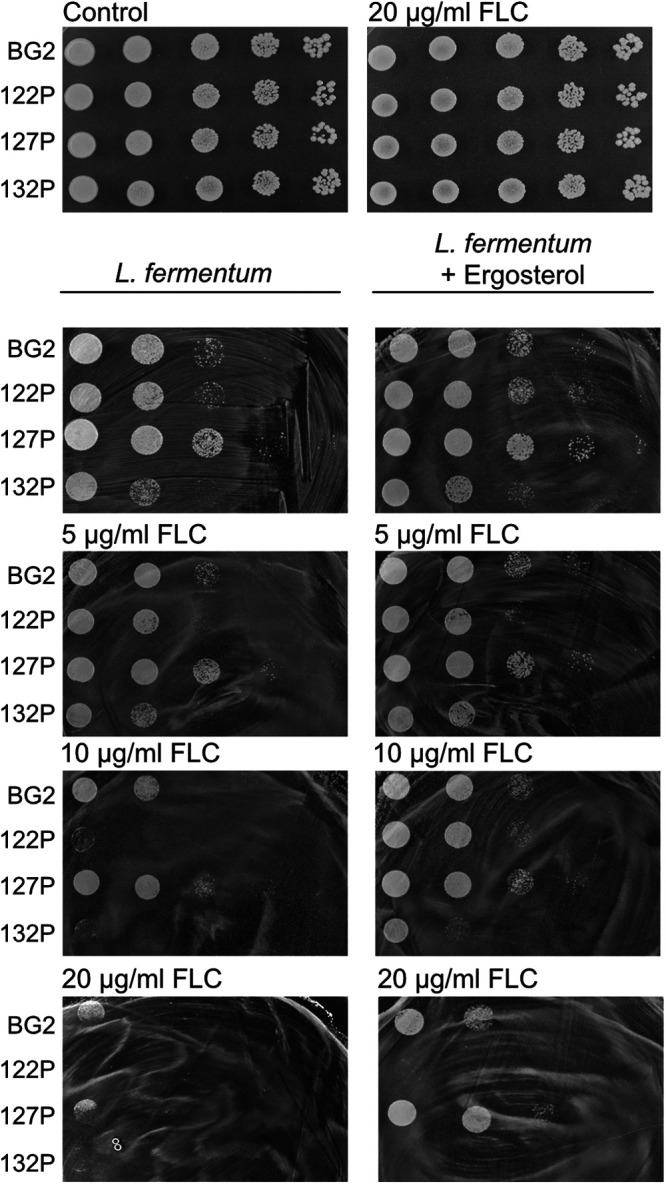
C. glabrata growth in coculture is dependent on ergosterol availability. C. glabrata strains were stressed with *L. fermentum* and fluconazole (FLC) with and without ergosterol (4.3 μg/mL) supplementation; isolates were spotted in serial dilutions (start OD_600_ of 1).

The above results suggested the possibility of a direct role of ergosterol in the *L. fermentum* C. glabrata coculture system. To test this possibility, we set up a coculture assay on solid medium. *L. fermentum* cells were spread out onto the plate, and C. glabrata was spotted onto the bacterial lawn. We added ergosterol and different concentrations of fluconazole to the medium (Fig. 5). C. glabrata isolates exposed to 5 μg/mL fluconazole in the presence of *L. fermentum* showed a slight decrease of growth compared with the control plate with only *L. fermentum*. Despite all isolates being resistant to 20 μg/mL fluconazole, a combination of 20 μg/mL fluconazole and *L. fermentum* abolished the growth of 122P and 132P and significantly reduced the growth of BG2 and 127P (Fig. 5). Fluconazole at a concentration of 10 μg/mL was sufficient to reduce the growth of 122P and 132P which are more sensitive to *L. fermentum*. Interestingly, the addition of ergosterol reduced the antifungal effect of *L. fermentum*.

**FIG 6:**
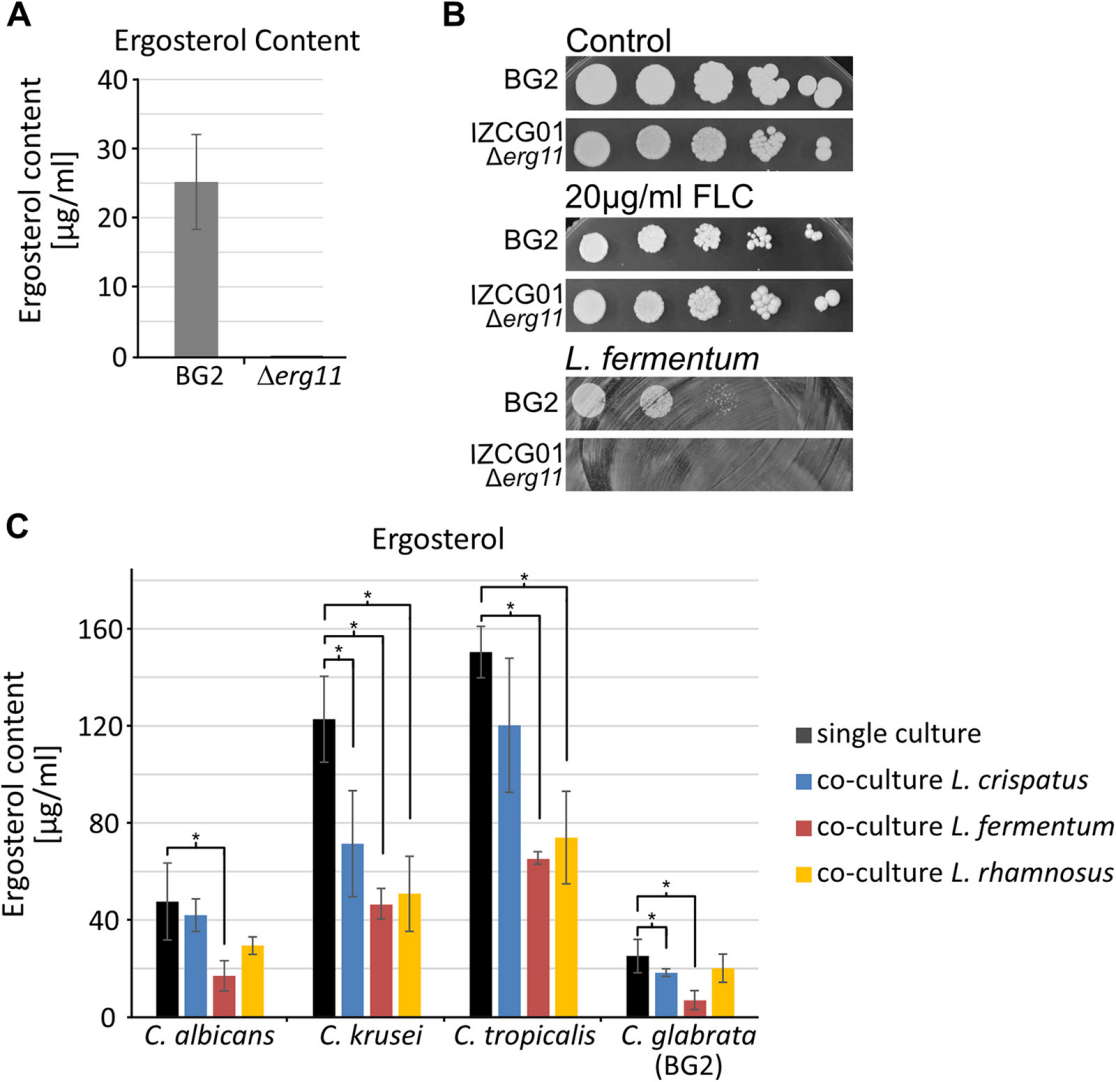
Ergosterol content during coculture. (A) Ergosterol content of BG2 and IZCG01 (*Δerg11*) in YPD. Data represent the average of at least two biological replicates (*n* = 2 to 3). (B) Serial dilutions of BG2 and IZCG01 (Δ*erg11*) on 20 μg/mL FLC or *L. fermentum* (50 μL; OD_600_ of 1). (C) Measurement of the ergosterol content of single cultures of C. albicans, C. krusei, C. tropicalis, and C. glabrata (BG2) and the respective *Candida* + *L. fermentum*/L. crispatus/L. rhamnosus GG cocultures after 10 h of incubation. Data represent the mean of at least 3 biological replicates (*n* = 3 to 6). Asterisks represent a statistical difference between single and coculture conditions (*, *P* ≤ 0.05).

If ergosterol is a key substance for the inhibition of C. glabrata and reduced ergosterol levels due to fluconazole treatment are the cause for more effective inhibition by *L. fermentum*, an ergosterol depletion by the interruption of its biosynthesis should display a similar phenotype. We thus deleted the *ERG11* gene encoding Sterol 14-demethylase from C. glabrata in the BG2 background. We confirmed the knockout mutant with standard methods and show with a chemical analytical approach using a high-pressure liquid chromatography (HPLC)-based method (see Materials and Methods section) that the Δ*erg11* mutant strain does not produce ergosterol (Fig. 6A). We found that this strain is unable to grow in the presence of *L. fermentum* but remains viable during fluconazole treatment (Fig. 6B). This result is in accordance with previous studies where *ERG11* knockout strains as well as a clinical isolate harboring an *ERG11* mutation showed resistance toward azoles (42, 43). Next, we asked if the ergosterol reduction is specific for the C. glabrata-*L. fermentum* coculture or occurs in other settings besides the already-described C. albicans-L. rhamnosus GG coculture (23, 24). We grew different *Candida* strains (C. albicans, Candida tropicalis, Candida krusei, and C. glabrata) in single-culture and coculture with different *Lactobacilli* species (L. rhamnosus and L. crispatus) and determined the ergosterol content of *Candida* cells (Fig. 6C). Importantly, we observed a significant depletion of fungal ergosterol in *L. fermentum* coculture across all tested *Candida* species. L. crispatus, a common vaginal *Lactobacillus* strain, significantly reduces ergosterol only in C. krusei and C. glabrata, whereas L. rhamnosus, a strain often used in probiotic formulations (25, 44), was able to decrease ergosterol content only in C. krusei and C. tropicalis. We conclude that the presence of *L. fermentum* reduces C. glabrata ergosterol content, which probably triggers the upregulation of steroid metabolism and limits fungal growth, and that this effect is common, albeit to a various degree in other *Candida*-*Lactobacillus* coculture settings.

## DISCUSSION

The effect of *Lactobacillus* species on fungi is manifold and dependent on many parameters, such as species, isolate, environment, and time. To describe the general and specific aspects of these interactions, we defined quantitative *in vitro* coculture conditions, isolated highly and less sensitive C. glabrata strains, and selected as a suitable *Lactobacillus* strain Limosilactobacillus fermentum (former Lactobacillus fermentum). Previous studies report on the different antifungal capacities of *Lactobacillus* species against *Candida* spp. (23, 24, 45, 46). Differences in antifungal activity could be due to the various ability of different lactobacilli isolates to produce metabolic by-products like lactic acid, acetic acid, or H_2_O_2_. However, the production capacity of lactic acid and H_2_O_2_ of lactobacilli does not correlate with the effectiveness against *Candida* species (45). Phenotyping of C. glabrata isolates did not reveal a significant correlation of stress sensitivity and growth inhibition by lactobacilli. Thus, the fungistatic effect of lactobacilli remains unclear.

To narrow down the mechanism of C. glabrata growth inhibition, we defined the transcriptomic response of C. glabrata when it was grown with *L. fermentum*. The coculture condition lead to a decrease of the adhesion-regulating gene *YAK1* and the Yak1-dependent adhesin *EPA6* in C. glabrata (see Table S1 in the supplemental material) in accordance with Chew (27). *Lactobacillus* spp. produce 1-acetyl β- carboline (1-ABC) which is linked to a reduction of filamentation via inhibition of YAK1 in C. albicans (47). Treatment of C. albicans with 1-ABC is not accompanied with a reduction in viability. Therefore, we reasoned that 1-ABC is not the reason for the antifungal effect of *L. fermentum* against C. glabrata. We found a gene expression pattern pointing at the involvement of an antifungal drug response pathway (Fig. 4A). Some antifungals (azoles) target the fungal plasma membrane containing ergosterol and sphingolipids (48). In accordance with that information, we found an upregulation of ergosterol biosynthesis genes in all isolates (Fig. S2). Previous studies reported that coculture of C. albicans and L. rhamnosus GG leads to a decrease in ergosterol content in the plasma membrane, as well as a downregulation of ergosterol biosynthesis (23, 24). Mailänder-Sánchez (24) analyzed the ergosterol content of the C. albicans and L. rhamnosus GG coculture on epithelial cells. In contrast to the findings of Mailänder-Sánchez, in our experimental setting, a coculture with L. rhamnosus did not lead to a significant ergosterol reduction in C. albicans but did in C. tropicalis and C. krusei. The reduction of ergosterol in the *Candida* cell membrane varied between different *Lactobacillus* species. Coculture with *L. fermentum* was the most effective, leading to a dramatic decrease of ergosterol in the fungal cell membrane of C. glabrata, as well as all other tested *Candida* species (Fig. 6C). We assume that this effect is probably triggering the upregulation of ergosterol synthesis. If ergosterol plays a direct role, a strain with compromised biosynthesis would possibly be hypersensitive to *L. fermentum* exposure. We generated a *Δerg11* strain in the BG2 strain background. The *Δerg11* strain, which cannot produce ergosterol, was indeed unable to grow in the presence of *L. fermentum. L. fermentum* is able to metabolize cholesterol (20), which is structurally similar to ergosterol. If *L. fermentum* is able to metabolize ergosterol too, it could lead to the decrease in ergosterol content during coculture.

In C. albicans, lactate as a carbon source leads to a lower expression of *CaERG11* and *CaERG3* and is accompanied by a reduction of ergosterol content in the plasma membrane (49). Lactobacilli in general produce lactic acid during homolactic fermentation (50). *ERG11* encodes a key enzyme of the ergosterol pathway and is regulated according to the demand of ergosterol biosynthesis. We found that *CgERG11* was upregulated during coculture but transiently downregulated in the presence of lactic acid after 1 h of treatment and returned to normal after 4 h (Fig. 4D). Therefore, in C. glabrata, lactic acid is unlikely a main player in the *L. fermentum*-dependent reduction of ergosterol. It is possible that C. albicans, unlike C. glabrata, lacks the response to compensate for *Lactobacillus* -induced downregulation of ergosterol synthesis and ergosterol content. This finding could explain the higher susceptibility of C. albicans isolates (compared with that of C. glabrata) to the fungistatic effect of *Lactobacillus* spp.

C. glabrata gene response pattern in coculture with *L. fermentum* shares similarities to mild FLC stress (51), hinting at a similar response pathway for both conditions. Strains BG2 and 127P had an MIC_50_ of 32 μg/mL to fluconazole, whereas 122P and 132P had lower MIC_50_ values (16 μg/mL and 8 μg/mL, respectively). BG2 is a clinical isolate known to be unresponsive to FLC treatment (29). FLC-resistant isolates BG2 and 127P were also more resistant to *L. fermentum*, which further hints to an overlap of fluconazole and *L. fermentum* response. Previous studies report that a combination of *Lactobacillus* and azole treatment was more effective at reducing C. albicans and C. glabrata burden than azole treatment alone in *in vivo* and *in vitro* (52–54). We confirm that the combination of fluconazole and *L. fermentum* decreases the growth of C. glabrata much more efficiently than fluconazole or lactobacilli treatment alone. Lourenço et al. (55) showed that 60 mM acetic acid at a low pH reduces resistance against FLC. The production of acetate was one of the main differences between effective and ineffective lactobacilli; however, concentrations around 4.6 ± 0.5 mM were measured (28). Compared with the data of Lourenço et al. (55), it would be too low to exert the combinational effect of acetic acid and FLC toward C. glabrata. In conclusion, we suggest that acetic acid does not play a major role in the combinational effect of FLC and lactobacilli. Taken together, the unavailability of ergosterol, either by fluconazole blocking ergosterol synthesis or by mutation, makes C. glabrata isolates more susceptible toward the effects of *L. fermentum*.

If ergosterol deprivation is a key determinant of C. glabrata inhibition by *L. fermentum*, the supplementation of the medium with ergosterol would likely support C. glabrata growth, which was indeed the case. C. glabrata can import sterols under aerobic and anaerobic conditions (56). It was shown that the presence of sterol can mitigate the effect of fluconazole in sterol-synthesis-defective mutants (38). The sterol transporter protein CgAus1 mediates sterol uptake (57). In addition, *ERG25* is needed for proper sterol uptake and helps to stabilize sterol-rich lipid domains in the cell membrane (58). However, in an aerobic environment, C. glabrata takes up sterols only during iron limitation conditions (57). It was also shown that iron limitation but not fluconazole stress leads to upregulation of *CgAUS1* (57). Furthermore, iron availability was linked to lower Erg11 activity and may subsequently reduce ergosterol content (59). We show here that the presence of *L. fermentum* leads to the upregulation of *CgAUS1* in all four tested isolates, as well as reduced ergosterol content. Additionally, genes regulated by Aft2, a transcription factor responsible for iron homeostasis in S. cerevisiae, were found upregulated under coculture conditions (Fig. 3A) (60). This result could hint at an involvement of the iron homeostasis system during coculture, which enables the import of sterols.

**FIG 7:**
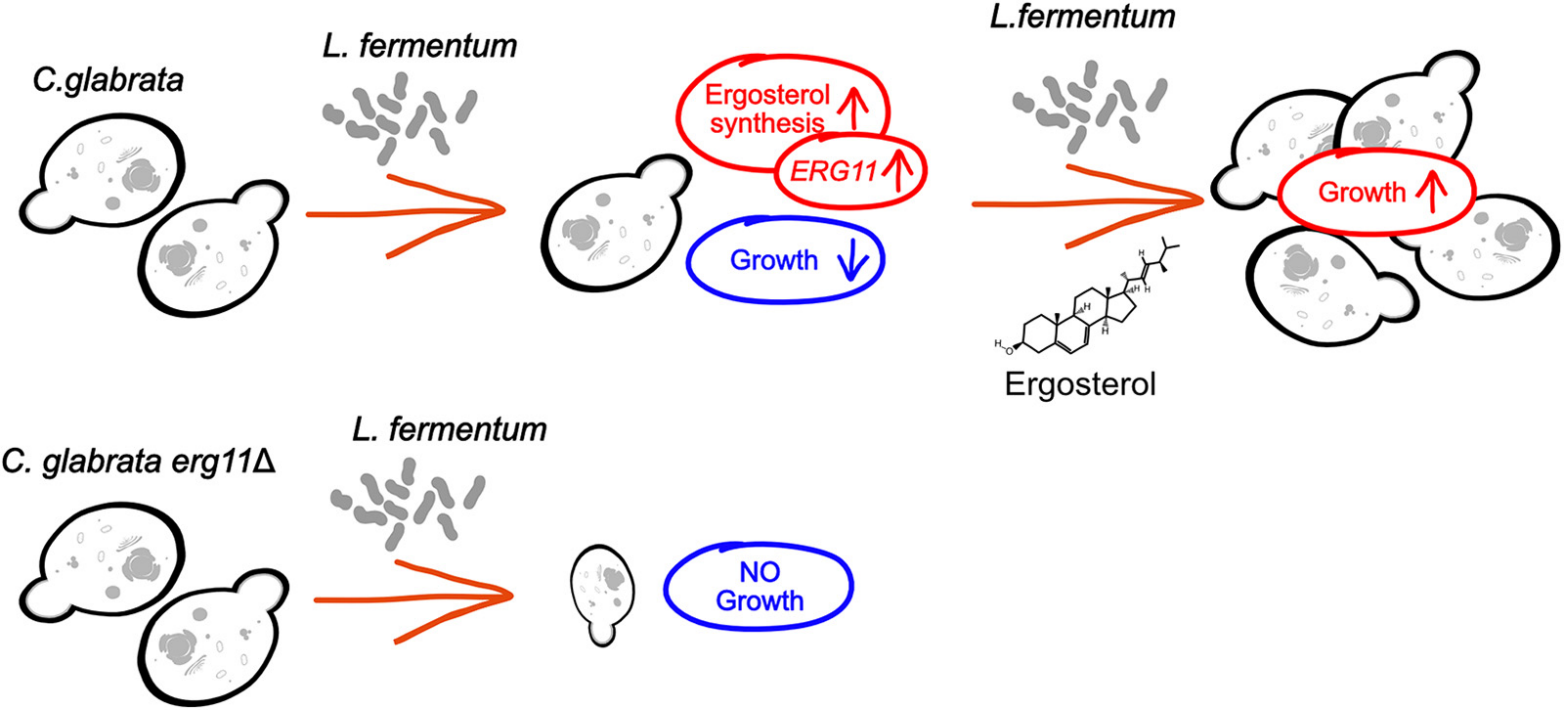
Schematic overview of the interaction between *L. fermentum* and C. glabrata. *Lactobacillus* leads to reduced growth, due to a decrease of ergosterol availability for C. glabrata.

The antifungal effect of *Lactobacillus* spp. toward C. glabrata is not fully understood. It is currently unclear what leads to a depletion of ergosterol and subsequent growth inhibition of C. glabrata if ergosterol cannot be replenished. The antifungal effect of *L. fermentum* varies substantially between different C. glabrata isolates. The common transcriptional response of the different isolates clearly revealed steroid biosynthesis as a key factor. However, the strain-specific transcriptional patterns did not allow us to deduce the basis of individual phenotypic differences. A further thorough exploration of the genomes of our *Candida* isolates could help to understand the genetic basis of the observed phenotypic differences. To our knowledge, we are the first to show that interactions between *L. fermentum* and C. glabrata lead to a lower ergosterol content in the fungal cell and upregulation of genes for sterol biosynthesis and import. The antifungal effect of *L. fermentum* can be enhanced by blocking ergosterol biosynthesis with azoles and reduced by ergosterol supplementation (Fig. 7). Finally, we regard the ergosterol depletion effect of *Candida* by *Lactobacillus* as strikingly common. These results suggest direct communication between the two species, and it is interesting to note that the bacterium is exploiting the same fungal molecular Achilles heel as conventional antifungal drugs.

## MATERIALS AND METHODS

### Microbial strains and culture conditions.

This study used the clinical isolate BG2 (29) and 93 clinical C. glabrata isolates, as well as C. albicans, C. krusei, and C. tropicalis isolates, which were collected and provided by the Institute of Hygiene and Microbiology at University Hospital St. Pölten. They were propagated in YPD medium at 37°C and 180 rpm. Limosilactobacillus fermentum and L. crispatus were collected and provided by the General Hospital of Vienna. L. rhamnosus (ATCC 53103) was purchased. Standard culture was performed in MRS medium at 37°C and 80 rpm.

### Strain construction.

A BG2 strain deleted for *ERG11* (IZCG01 Δ*erg11*::*NAT1*) was generated. Flanking regions (500 bp) of *ERG11* (CAGL0E04334g) were amplified from the genomic DNA of BG2. *NAT1* was amplified from plasmid pV1382 (Addgene plasmid number 111436; http://n2t.net/addgene number 111436; RRID:Addgene_111436) (61). The fragments were joined using the primer pair IZ1 and IZ4. The resulting fragment was transformed into BG2 via a heat shock method described previously (62) using ClonNAT (Sigma-Aldrich) as positive selection. Clones were screened for the correct insert using primers IZ_col_erg_F and IZ_col_erg_R.

A BG2 strain deleted for *PDR12* (IZGC02 Δ*pdr12*) was generated via CRISPR-Cas9 using the a method described previously (61). The plasmid pV1326 (Addgene plasmid number 111435; http://n2t.net/addgene number 111435; RRID:Addgene_111435) was used (61). A single guide RNA was designed using CHOPCHOP v3 (63). ClonNAT (Sigma-Aldrich) was used as the positive selection. All oligonucleotides used in this study are listed in Table 1.

**TABLE 1:** List of oligonucleotides used in this study

Name	Sequence (5′–3′)	Description
IZ1	CGATTGTATCGGACAAATCG	ERG11_flank1_F
IZ2	GACGAGGCAAGCTTGATGCCAAAATTGCAGTTTGTTAAGGG	ERG11_flank1_R + overhang NAT1
IZ3	GAT TTG ATA CTA ACG CCG CCA AGG TTC ATA GCC ATA TTC TGG	ERG11_flank2_F + overhang NAT1
IZ4	GGAAGATCATATTGAATCTGG	ERG11_flank2_R
IZ_col_erg_F	CGA CTA CTT CAA GGC TAT TTG	Colony PCR deltaERG11
IZ_col_erg_R	GTA AAC TTC GCC TCC AGA AA	Colony PCR deltaERG11
IZ_nat_F	CTT GGC GGC GTT AGT ATC AAA TCG	NAT1 gene amplification
IZ_nat_R	CAA AGA GCG GCC GCA TCA AGC TTG	NAT1 gene amplification
pdr12_guide1_F	*GAT CGA GTT ATC TCC ACC AAG ACA AG*	Single guide RNA for CRISPR/Cas
pdr12_guide1_R	AAA ACT TGT CTT GGT GGA GAT AAC TC	Single guide RNA for CRISPR-Cas
pdr12_check1_F	AGC AAT GTT GAG CAA CCA GC	Colony PCR deltaPDR12
pdr12_check1_R	AAG GAA AGG AAG GAT GAT GC-	Colony PCR deltaPDR12

### Liquid coculture assay.

An overnight culture of C. glabrata was diluted to an optical density at 600 nm (OD_600_) of 0.1 into MRS containing *L. fermentum* at an OD_600_ of 0.05. Single cultures with only *Candida* were used as the control. Cultures were incubated at 37°C and 80 rpm for 10 h. Serial dilutions were plated onto YPD supplemented with 50 μg/mL ampicillin. CFUs were counted the next day, and log CFU/mL was calculated.

### Spotting assay.

Overnight cultures of C. glabrata were regrown until an OD_600_ of 1. Spots (2 μL) of a serial dilution (1:10) were spotted onto MRS, onto which 50 μL of *L. fermentum* (OD_600_ of 1) was spread previously. Up to 20 μg/ml Fluconazole was added to the MRS medium. For ergosterol supplementation, 4 μg/ml ergosterol (Sigma-Aldrich) was added to the medium. Plates were incubated and inspected every day for up to 6 days.

### RNA extraction.

RNA extraction was done by glass bead disruption and phenol-chloroform extraction as described earlier from a 30-mL culture in 200 μL extraction buffer (50 mM Tris [pH 7], 130 mM NaCl, 5 mM EDTA, and 5% SDS) and 200 μL phenol-chloroform-isoamylalcohol (25:24:1; Carl Roth GmbH, Germany). The upper aqueous layer was extracted twice with chloroform-isoamylalcohol (24:1), and the RNA was precipitated with EtOH/NaAcetate, washed and dried, and resuspended in 50 μL RNase-free water.

### Library preparation, sequencing, and differential gene expression analysis.

Library preparation and sequencing were performed by the Next Generation Sequencing Facility at Vienna BioCenter Core Facilities (VBCF), a member of the Vienna BioCenter (VBC), Austria. PolyA enrichment library preparation and single-read sequencing (50 bp, Illumina HiSeqV4) were obtained of BG2, 122P, 127P, and 132P with and without *L. fermentum*. At least 2 biological replications of each sample were sequenced and used for subsequent analysis. Demultiplexing was performed by VBCF NGS with bcl2fastq v2.20.0.422. Quality control was performed using FastQC. Reads were aligned against the C. glabrata CBS138 reference genome, which was obtained from the *Candida* Genome Database (CGD; http://www.candidagenome.org), using Bowtie 2 (64). Quantification of the aligned reads was performed with Rsubread (65) with default settings. Identification of differentially expressed genes (DEGs) between single culture and coculture of each isolate was done using R package DESeq2 (66) with default parameters. Arbitrary cutoff values for DEGs (*P* ≤ 0.05 and log_2_ fold change of ≥1.5) were chosen. GO term enrichment analysis for the category “metabolic process” was performed using the database of CGD with default settings. Clustering according to transcription factor (TF) was performed using the database of PathoYeastract (http://pathoyeastract.org). Only documented entries were used for “search by TF.”

### Phenotypic stress test and MIC assay.

Overnight cultures of C. glabrata were regrown in YPD, SD full (yeast nitrogen base without amino acids supplemented with ammonium sulfate; BD Difco USA), or MRS medium up to an OD_600_ of ~0.5 and diluted 1:10 into the respective medium in a 96-well flat-bottom plate. Stress tests with l-lactic acid (Carl Roth GmbH, Germany), acetic acid (Carl Roth GmbH), H_2_O_2_ (Carl Roth GmbH), and at pH 4 and pH 8 were done in SD full medium. For lactic acid and acetic acid, pH was adjusted to pH 4 with HCl; 2% l-lactate (wt/vol) as a carbon source was used in SD full medium, with the pH adjusted to pH 4 with HCl. For every tested condition, C. glabrata isolates were incubated at 37°C in at least triplicates and OD_600_ was monitored with an automated set-up (Cytomat42 [Thermo Fisher Scientific, MA, USA], Synergy 95 H1 [Agilent, USA], and Rack Runner 720 [Hamilton Robotics, Germany]). An MIC assay with FLC (0.125 to 256 mg/L) was performed in SD full medium. C. glabrata cells were inoculated with an OD_600_ of ~0.05 and incubated at 37°C. The OD_600_ was monitored in 2-h intervals for 24 h with a fully automated set-up.

### Ergosterol quantification.

For the extraction of ergosterol content, the isolates were grown in MRS until an OD_600_ of 40 was reached. Coculture samples were separated by centrifugation (2,000 rpm for 5 min). The quantification method was described previously (67). In short, pellets were resuspended with 250 μL 10% (wt/wt) KOH in MeOH, followed by sonication (15 min) and 70°C for 50 min. A total of 50 μL HPLC-grade water and 100 μL n-hexane (Merck, Germany) were added, and the phases were separated by centrifugation. The hydrophobic upper phase was collected followed by a second extraction step. n-Hexane was evaporated at 40°C. When it was completely dry, 100 μL MeOH (Merck, Germany) was added and incubated for 15 min at 40°C. Ergosterol was determined via C_18_ reverse phase using HPLC (1200 Series; Agilent Technologies, USA), and detection was performed at 282 nm, a column temperature of 25°C, an isocratic elution of 95% MeOH, and a 5% H_2_O flow rate of 0.3 mL min^−1^. The retention time of ergosterol was 3.9 min. Ergosterol content was calculated using a standard curve. Ergosterol content is stated as μg per mL extraction volume.

### Statistical analysis.

Growth rates for the MIC assay and phenotypic stress tests were calculated using the “growthcurver” package in R (68, 69). A heatmap for Fig. 2 was prepared using cluster 3.0 (70) and Java Treeview (71). Clustering for this heatmap was done using complete linkage clustering and correlation as a similarity metric. Other heatmaps were generated using the R package “pheatmap.” The genes used for diverse heatmaps are listed in Table S2 in the supplemental material. The principal component analysis was performed and displayed using DESeq2. Dot plots were generated using ggplot2 (72).

### Data availability.

Raw RNA sequencing data were uploaded to the Gene Expression Omnibus (GEO) under accession number GSE202656.
