# Supplementary material for: Limosilactobacillus fermentum Limits Candida glabrata Growth by Ergosterol Depletion
Source: Microbiol Spectr. 2023 Feb 21;11(2):e03326-22. doi: 10.1128/spectrum.03326-22 (PMC10100998; doi:10.1128/spectrum.03326-22)
Supplement: Supplemental file 5 — Supplemental material. Download spectrum.03326-22-s0005.pdf, PDF file, 0.8 MB [file spectrum.03326-22-s0005.pdf]

SUPPLEMENTARY DATA

Figure S1 *Heatmap comparison of different datasets;* (A) Heatmap of expression data of acetate as carbon source compared to *Lactobacillus* response of the tested isolates; (B) Heatmap of expression data of genes regulated by Haa1 compared to *Lactobacillus* response of the tested isolates; (C) Heatmap of expression data of oxidative stress compared to *Lactobacillus* response of the tested isolates; Colour code: upregulation red, downregulation blue

Figure S2 *Ergosterol Biosynthesis is upregulated during co-culture* Heatmap of the genes involved in Ergosterol Biosynthesis; data represents the log2FC of each gene;

Figure S3 *Involvement of oxygen availability during co-culture* (A) Oxygen percentage of medium of co-culture and single *Candida* culture after 10h of incubation; measurement was done with three biological replicates (n=3); (B) Heatmap of gene expression data of anaerobic upregulated genes in *S. cerevisiae* compared to *Lactobacillus* response in *C. glabrata*; upregulation red, downregulation blue

Table S1 Log2 Fold-change data of each gene and sample

Table S2 Genes designations for heatmaps

Table S3 Values used for figures 1AC, 4BCD, 6AB

Table S4 Gene designations for the heatmap in Figure 2C and GO Terms according to CGD for the different clusters a-f

Figure S1

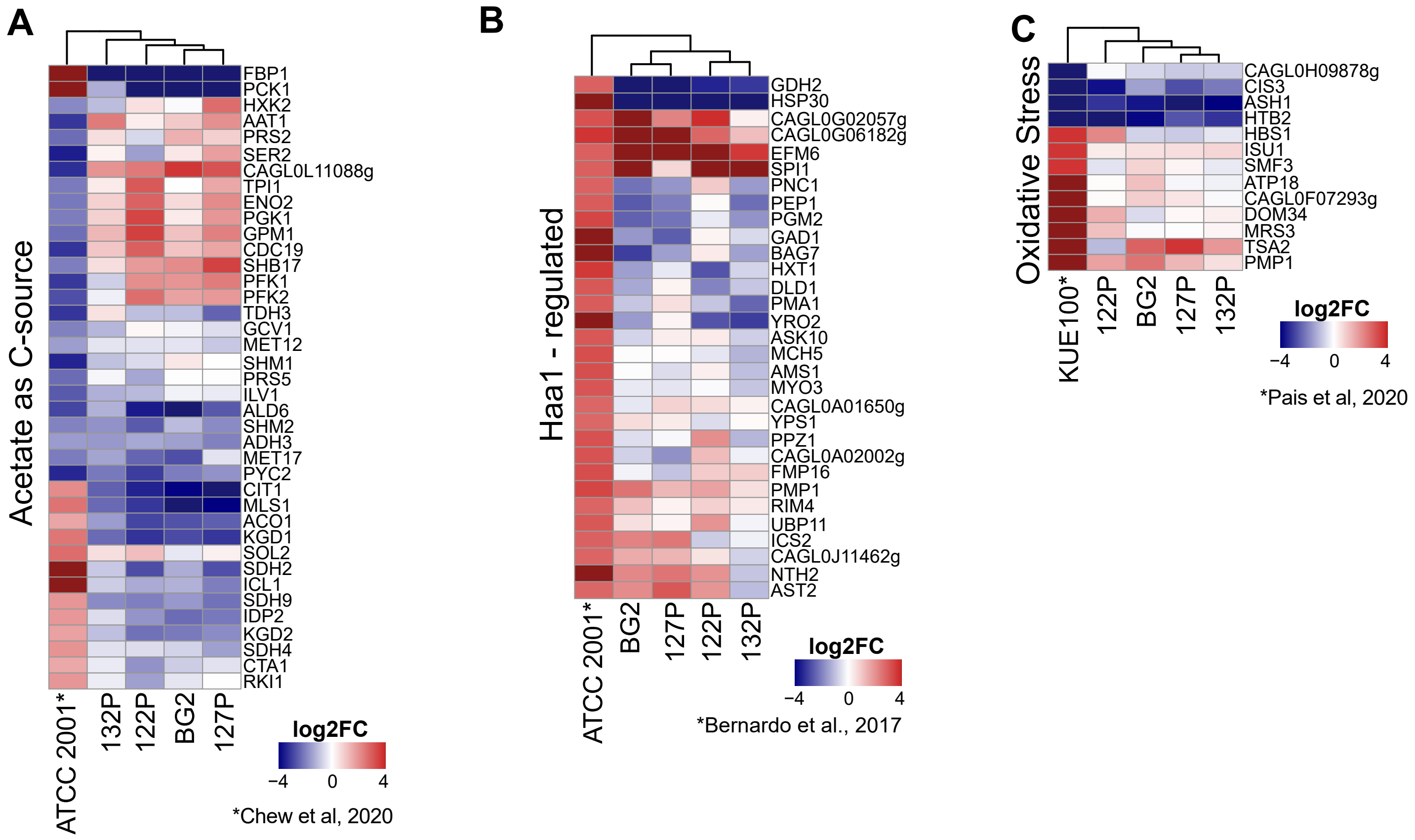
A
Acetate as C-source
FBP1
PCK1
HXK2
AAT1
PRS2
SER2
CAGL0L11088g
TPI1
ENO2
PGK1
GPM1
CDC19
SHB17
PFK1
PFK2
TDH3
GCV1
MET12
SHM1
PRS5
ILV1
ALD6
SHM2
ADH3
MET17
PYC2
CIT1
MLS1
ACO1
KGD1
SOL2
SDH2
ICL1
SDH9
IDP2
KGD2
SDH4
CTA1
RKI1
ATCC 2001*
132P
122P
BG2
127P
log2FC
−4
0
4
*Chew et al, 2020
B
Haa1 - regulated
GDH2
HSP30
CAGL0G02057g
CAGL0G06182g
EFM6
SPI1
PNC1
PEP1
PGM2
GAD1
BAG7
HXT1
DLD1
PMA1
YRO2
ASK10
MCH5
AMS1
MYO3
CAGL0A01650g
YPS1
PPZ1
CAGL0A02002g
FMP16
PMP1
RIM4
UBP11
ICS2
CAGL0J11462g
NTH2
AST2
ATCC 2001*
BG2
127P
122P
132P
log2FC
−4
0
4
*Bernardo et al., 2017
C
Oxidative Stress
CAGL0H09878g
CIS3
ASH1
HTB2
HBS1
ISU1
SMF3
ATP18
CAGL0F07293g
DOM34
MRS3
TSA2
PMP1
KUE100*
122P
BG2
127P
132P
log2FC
−4
0
4
*Pais et al, 2020

Figure S2

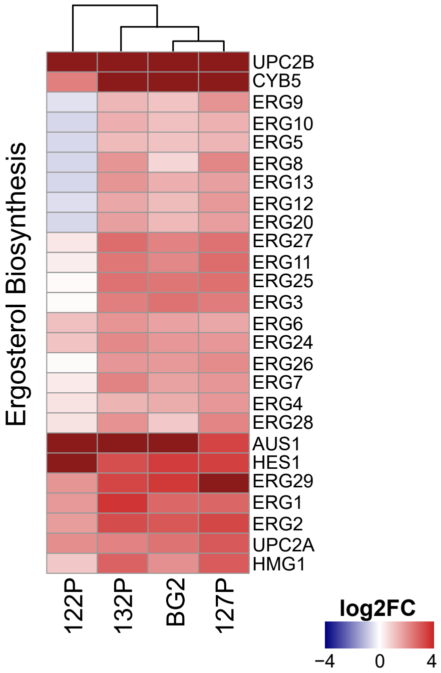

Ergosterol Biosynthesis
UPC2B
CYB5
ERG9
ERG10
ERG5
ERG8
ERG13
ERG12
ERG20
ERG27
ERG11
ERG25
ERG3
ERG6
ERG24
ERG26
ERG7
ERG4
ERG28
AUS1
HES1
ERG29
ERG1
ERG2
UPC2A
HMG1
122P
132P
BG2
127P
log2FC
−4
0
4

Figure S3

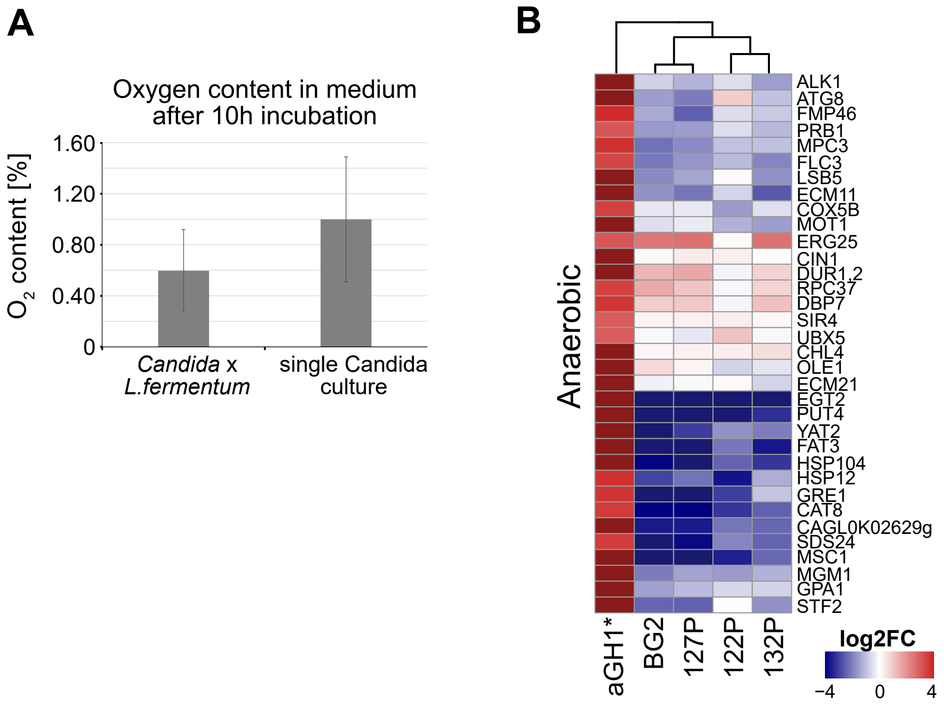

*Becerra et al, 2002
